# Rates of Influenza-like Illness and Winter School Breaks, Chile, 2004–2010

**DOI:** 10.3201/eid2007.130967

**Published:** 2014-07

**Authors:** Gerardo Chowell, Sherry Towers, Cécile Viboud, Rodrigo Fuentes, Viviana Sotomayor

**Affiliations:** Arizona State University, Tempe, Arizona, USA (G. Chowell, S. Towers);; National Institutes of Health, Bethesda, Maryland, USA (G. Chowell, C. Viboud);; Ministerio de Salud, Santiago, Chile (R. Fuentes, V. Sotomayor)

**Keywords:** Influenza-like illness, Chile, age-specific incidence rates, incidence ratio, influenza, viruses

## Abstract

To determine effects of school breaks on influenza virus transmission in the Southern Hemisphere, we analyzed 2004–2010 influenza-like–illness surveillance data from Chile. Winter breaks were significantly associated with a two-thirds temporary incidence reduction among schoolchildren, which supports use of school closure to temporarily reduce illness, especially among schoolchildren, in the Southern Hemisphere.

Influenza pandemic preparedness plans to mitigate effects of a severe pandemic recommend layered medical and social distancing interventions, including school closings, cancellation of large public gatherings, and face mask use (*1*). Because schoolchildren are considered to be high transmitters of influenza virus (higher contact rates, enhanced susceptibility to infection, and increased virus shedding relative to that among persons in other age groups), prompt school closure is expected to reduce transmission during a pandemic (*2*).

Although several empirical studies have linked school activities with influenza virus transmission (*2*–*10*), few studies have considered data from multiple epidemic periods, and little information is available from the Southern Hemisphere. School breaks and school teacher strikes provide natural experiments in which the effect of school terms on influenza transmission dynamics can be explored. On the basis of 21 years of surveillance data, Cauchemez et al. (*5*) found a 16%–18% reduction in incidence of influenza-like illness (ILI) associated with the 2-week school winter break periods in France. A study of variation in contact rate patterns in Europe suggested a 13%–40% reduction in the basic reproduction number associated with school breaks in Belgium, Great Britain, and the Netherlands (*11*). A 12-day teacher strike in Israel in the winter of 1999 was also associated with a reduction (43%) in weekly rates of respiratory disease (*12*). A single study is available from the Southern Hemisphere and indicates a 14% reduction in ILI incidence during winter break in Argentina during 2005–2008; the largest decrease was observed among children 5–14 years of age (*6*). In our study, we quantified the effect of school break cycles on the age distribution of ILI patients in Chile during 2004–2010.

## The Study

We obtained weekly age-specific ILI incidence rates during 2004–2010 from a systematic national surveillance system in Chile (*13*). ILI surveillance relies on 42 sentinel outpatient sites located throughout the country; these sites are representative of the general population and systematically report weekly age-specific physician visits for ILI (*13*) (online Technical Appendix, http://wwwnc.cdc.gov/EID/article/20/7/13-0967-Techapp1.pdf). We characterized the effect of the 2-week winter break period on influenza transmission during 2004–2010 by comparing trends in weekly ILI incidence rates among schoolchildren (5–14 and 15–19 years of age) and adults (20–64 and >65 years of age). To estimate changes in the age distribution of ILI patients, on the basis of methods used in previous work (*8*,*14*), we compared the weekly ratios of ILI incidence rates for schoolchildren and adults during the 2-week period before, during, and after the winter break by using a 1-sided Z test. We also considered a 6-week window (*8*,*14*) before and after the winter break as a sensitivity analysis. A decline in the schoolchildren-to-adult incidence rate ratio indicates a shift in the age distribution of patients toward adults, suggestive of decreased influenza transmission among schoolchildren (*8*).

In Chile, wintertime influenza activity peaks during May–September, which is typical of temperate regions in the Southern Hemisphere (*15*). The 2-week winter school break typically coincides with the influenza season and is synchronous throughout the country; ≈95% of educational institutions follow the break periods set by the Ministry of Education.

**Figure 1:**
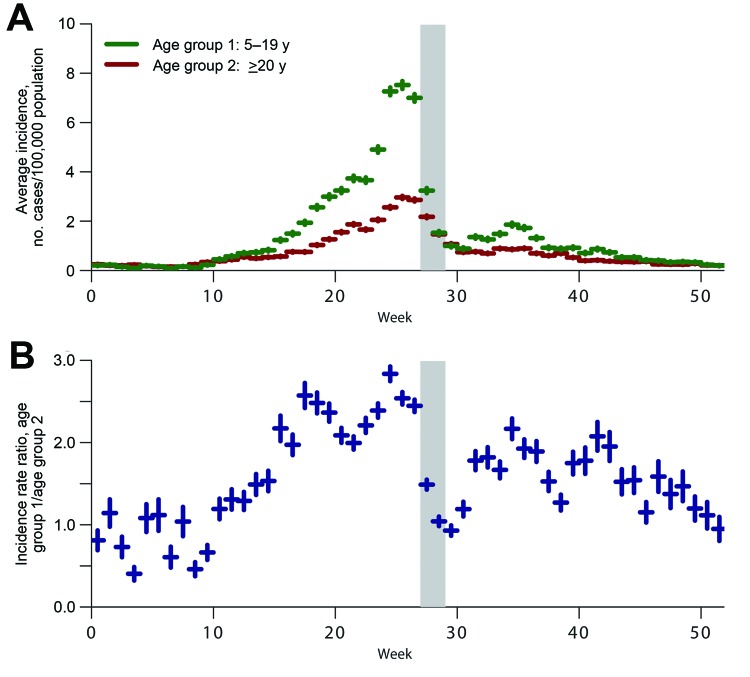
Average weekly incidence rates for influenza-like illness (ILI) among schoolchildren 5–19 years of age and adults >20 years of age, Chile, 2004–2010. Error bars represent the standard errors of the mean within each week. The shaded area represents the period of the 2-week winter break. A) Average ILI incidence per 100,000 population, by week. B) Average ILI incidence rate ratio of schoolchildren-to-adult incidence by week.  Examination of a 2-week period and comparison of the averaged within–week-of-year ILI incidence rate ratio for children (5–19 years of age) to adults (>20 years of age) to the average of the ratio in the 2-week period immediately before provided 50 such comparisons. The Bonferroni corrected α = 0.05; significance level is thus α = 0.05/50 = 0.001. The only 2-week periods in which the ratio comparison p value was less than α = 0.001 were the periods beginning week 28 and 29 (which corresponds to the winter break), week 44 (which corresponds to the Reformation/All Saints Day 4-day weekend), week 21 (the week of the Naval Glories Day break), and week 38 (the Independence Day break).

Figure 1 illustrates trends in ILI incidence rates among schoolchildren 5–19 years of age and adults >20 years of age throughout the year and the associated schoolchildren-to-adults incidence rate ratio. In Chile, ILI incidence displays bimodal patterns of activity; activity increases before and after the winter break, and transmission is reduced during the break. The schoolchildren-to-adults ratios decreased substantially (40%–68%) during the 2-week winter break period relative to the 2-week period immediately preceding the winter break (Table 1). Also, the reduction in ratios coinciding with the first week of the winter break occurred every year of our study, including during the pandemic (2009) and postpandemic (2010) seasons.

**Table 1:** ILI incidence rate ratios for schoolchildren to adults during 2-week periods surrounding school winter breaks, Chile, 2004–2010*

Age group, y	ILI incidence rate ratio				p value	
	Before school break	During school break	After school break		Before break vs. during break†	During break vs. after break‡
Adults >20						
Schoolchildren 5–14	2.68 (0.06)	1.28 (0.16)	1.88 (0.117)		<0.001	0.002
Schoolchildren 15–19	2.11 (0.12)	1.22 (0.04)	1.63 (0.14)		<0.001	0.002
Schoolchildren 5–19	2.49 (0.06)	1.26 (0.11)	1.80 (0.08		<0.001	<0.001
Adults 20–64						
Schoolchildren 5–14	2.52 (0.06)	1.25 (0.16)	1.92 (0.13)		<0.001	<0.001
Schoolchildren 15–19	1.98 (0.10)	1.18 (0.04)	1.66 (0.13)		<0.001	<0.001
Schoolchildren 5–19	2.34 (0.05)	1.23 (0.11)	1.83 (0.09)		<0.001	<0.001
Adults >65						
Schoolchildren 5–14	5.07 (0.48)	1.61 (0.23)	1.65 (0.16)		<0.001	0.451
Schoolchildren 15–19	4.01 (0.56)	1.52 (0.08)	1.44 (0.22)		<0.001	0.626
Schoolchildren 5–19	4.71 (0.50)	1.58 (0.16)	1.58 (0.17)		<0.001	0.505

*The “after break” period begins 2 weeks after the winter break ends because the reduction in the incidence rate ratio during the winter break was maintained on average for 2 weeks after the end of the winter break. ILI, influenza-like illness. †p value of a 1-sided Z test comparing the average incidence rate ratio (ratio of incidence rate for schoolchildren to incidence rate for adults) during the 2-week period before the school break to that during the winter break. Small p values indicate that the incidence rate ratio for the period before the break is significantly higher than that for the period during the break;. p values near 1.00 indicate that the incidence rate ratio for the period before the break is significantly lower than that for during the break. ‡p value of a 1-sided Z test comparing the average incidence rate ratio during the 2-week period after the school break to that during the winter break period. Small p values indicate that the incidence rate ratio for the period after the winter break period is significantly higher than that for the period during the winter break; p values near 1.00 indicate that the incidence ratio for the period after the break is significantly lower than that for the period during the break.

The reduction in the schoolchildren-to-adults incidence rate ratios was maintained for an average of 2 weeks after the end of the winter break. The decline in ratios was primarily caused by a decrease in ILI rates among schoolchildren; the average (+ standard error of the estimate) reduction in ILI incidence among schoolchildren (5–19 years of age) in the 2 weeks during the winter break compared with the 2 weeks before was 67.2% + 2.1% (p<0.001). This reduction occurred systematically in each winter of the study period. In contrast, the average reduction in adult ILI incidence (>20 years of age) was more modest but remained significant at 37.4% + 0.9% (p<0.001).

Furthermore, the incidence rate ratios for school-age children to middle-age adults significantly increased after the winter break, signaling a return toward a higher proportion of ILI cases among children, although the ratio did not return to prebreak levels (Table 1). In contrast, the ratio comparing rates for children with rates for adults did not change. Our results did not change when we used a 6-week period before and after the winter break period instead of a 2-week period (Table 2) or when we excluded the 2009 pandemic year from our analysis (Technical Appendix Figure 1).

**Table 2:** ILI incidence rate ratios for schoolchildren-to-adult age groups during 6-week periods surrounding school winter breaks, Chile, 2004–2010*

Age group, y	ILI incidence rate ratio				p value	
	Before school break	During school break	After school break		Before break vs. during break†	During break vs. after break‡
Adults >20						
Schoolchildren 5–14	2.59 (0.14)	1.28 (0.16)	1.98 (0.10)		<0.001	<0.001
Schoolchildren 15–19	2.02 (0.07)	1.22 (0.04)	1.66 (0.07)		<0.001	<0.001
Schoolchildren 5–19	2.40 (0.11)	1.26 (0.11)	1.87 (0.07)		<0.001	<0.001
Adults 20–64						
Schoolchildren 5–14	2.52 (0.11)	1.25 (0.16)	2.00 (0.10)		<0.001	<0.001
Schoolchildren 15–19	1.97 (0.05)	1.18 (0.04)	1.68 (0.07)		<0.001	<0.001
Schoolchildren 5–19	2.33 (0.08)	1.23 (0.11)	1.89 (0.08)		<0.001	<0.001
Adults >65						
Schoolchildren 5–14	3.82 (0.62)	1.61 (0.23)	1.85 (0.14)		<0.001	0.187
Schoolchildren 15–19	2.94 (0.46)	1.52 (0.08)	1.56 (0.11)		0.001	0.401
Schoolchildren 5–19	3.52 (0.56)	1.58 (0.16)	1.75 (0.12)		<0.001	0.206

*The “after break” period begins 2 weeks after the winter break ends because the reduction in the incidence rate ratio during the winter break was maintained on average for 2 weeks after the end of the winter break. ILI, influenza-like illness. †p value of a 1-sided Z test comparing the average incidence rate ratio (ratio of incidence rate for schoolchildren to incidence rate for adults) during the 2-week period before the school break to that during the winter break. Small p values indicate that the incidence rate ratio for the period before the break is significantly higher than that for the period during the break; p values near 1.00 indicate that the incidence rate ratio for the period before the break is significantly lower than that for during the break. ‡p value of a 1-sided Z test comparing the average incidence rate ratio during the 2-week period after the school break to that during the winter break period. Small p values indicate that the incidence rate ratio for the period after the winter break period is significantly higher than that for the period during the winter break; p values near 1.00 indicate that the incidence rate ratio for the period after the break is significantly lower than that for the period during the break.

## Conclusions

We have shown that a two-thirds decline in ILI incidence among schoolchildren coincided with the onset of the school winter break in Chile; this pattern was consistent across the 7 years of the study. In line with a prior study in Argentina (*6*), the average reduction in schoolchildren-to-adults incidence rate ratio was sustained for up to 2 weeks after school sessions resumed. This time scale is consistent with the natural history of influenza virus infection, which has a serial interval (interval between cases) of 2–3 days, so that it takes a few successive chains of transmission to reach full-scale transmission.

**Figure 2:**
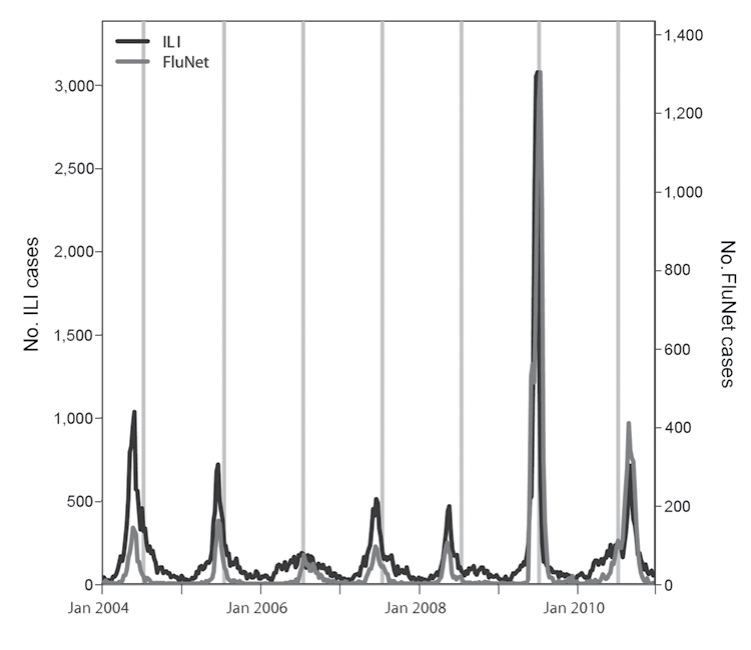
Weekly time series of influenza-like-illness (ILI) cases and laboratory-confirmed influenza notifications (FluNet [*15*]), in Chile, 2004–2010. The shaded areas represent the 2-week winter break periods.

Similar to findings from prior studies (*5*,*6*), our findings are based on analysis of ILI incidence, which is a broad indicator of respiratory disease activity in a community and is not entirely specific for influenza. Our results could be affected by changes in health-seeking behavior during the winter break. However, our ILI data are well correlated with influenza virus activity data (*15*) (Figure 2), and large increases in incidence among schoolchildren during winter 2009 coincide with the influenza A(H1N1)pdm09 virus pandemic period, suggesting that fluctuations in ILI incidence in Chile are primarily attributable to influenza. Our data also support the conclusion that school closure during pandemic situations is effective. Although the winter break took place near the peak of the 2009 influenza A(H1N1) pandemic in Chile, it was correlated with changes in the age distribution of patients hospitalized for influenza A(H1N1)pdm09 virus infection (*7*).

Overall, our study findings add to the body of information provided by empirical studies, supporting the implementation of school closure to achieve temporary reductions in ILI incidence rates, especially among school-age children, including in the Southern Hemisphere temperate setting (*2*–*7*). Our finding that ILI incidence was more modestly reduced among adults during winter breaks is consistent with past work on the age-specific transmission dynamics of influenza (*2*–*7*). School closure may be particularly useful in pandemic situations to gain time until pharmaceutical measures (vaccines, antiviral medications) become available and to mitigate the burden on health care institutions by reducing the surge of influenza patients. There is still, however, little information available from tropical and Southern Hemisphere settings, which are characterized by complex influenza seasonality patterns and/or low connectivity with the rest of the world and particular demographic and health conditions. Systematic multicountry and multiyear comparison of the effects of school closures could shed light on the effectiveness of school-based intervention policies under different epidemiologic, behavioral, and demographic situations.

Technical Appendix Epidemiological surveillance of influenza-like illness (ILI) in Chile. Average weekly incidence rates for ILI among schoolchildren 5–15 years of age and adults >20 years of age, Chile, 2004–2010, excluding data from the 2009 influenza A(H1N1) pandemic year.
