# Supplementary material for: Rates of Influenza-like Illness and Winter School Breaks, Chile, 2004–2010
Source: Emerg Infect Dis. 2014 Jul;20(7):1195–9. doi: 10.3201/eid2007.130967 (PMC4073857; doi:10.3201/eid2007.130967)
Supplement: Technical Appendix — Epidemiological surveillance of influenza-like illness (ILI) in Chile. Average weekly incidence rates for ILI among schoolchildren 5–15 years of age and adults >20 years of age, Chile, 2004–2010, excluding data from the 2009 influenza A(H1N1) pandemic year. [file 13-0967-Techapp-s1.pdf]

DOI: http://dx.doi.org/10.3201/eid2007.130967

Rates of Influenza-like Illness and Winter School Breaks, Chile, 2004–2010

Technical Appendix

Epidemiological Surveillance of Influenza-like Illness in Chile

Importantly, the surveillance data for ILI in Chile is representative of the general population (*1*). Specifically, epidemiological surveillance for influenza-like-illness in Chile is carried out by a number of sentinel sites (n = 42) that capture a substantial amount of ILI outpatient visits throughout Chile. These sentinel sites provide medical assistance to a well-defined population of ≈1.3 million, cover primary medical assistance to all age groups, and have a motivated team to support epidemiological surveillance. Importantly, these sentinel sites have a registered population which permits the estimation of ILI incidence rates. Each sentinel site must notify weekly ILI case counts according to age groups and gender to regional authorities that subsequently report these data to the Ministry of Health (*1*).

Reference

1. Ministerio de Salud de Chile, Departamento de Epidemiologia. Vigilancia epidemiológica, investigación y control de brotes [cited 2013 Jun 4]. http://epi.minsal.cl/epi/html/normas/circul/CircularInfluenzaESTACIONALyPANDEMICA.pdf

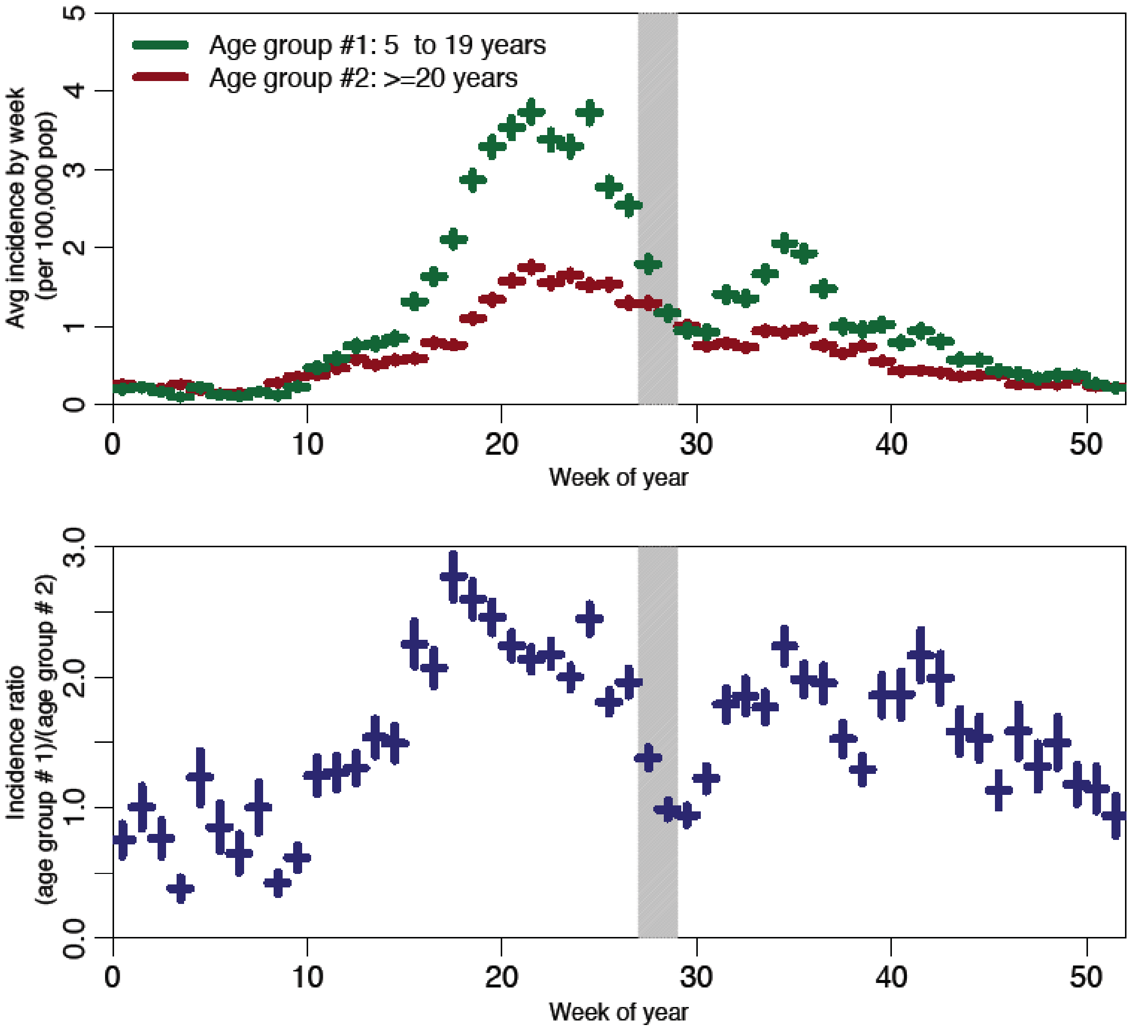

Figure. Average weekly incidence rates for influenza-like illness (ILI) among schoolchildren 5–19 years of age and adults >20 years of age, Chile, 2004–2010,excluding data from the 2009 A/H1N1 influenza pandemic year.
